# Stroma and lymphocytes identified by deep learning are independent predictors for survival in pancreatic cancer

**DOI:** 10.1038/s41598-025-94362-x

**Published:** 2025-03-19

**Authors:** Xiuxiang Tan, Mika Rosin, Simone Appinger, Julia Campello Deierl, Konrad Reichel, Mariëlle Coolsen, Liselot Valkenburg-van Iersel, Judith de Vos-Geelen, Evelien J. M. de Jong, Jan Bednarsch, Bas Grootkoerkamp, Michail Doukas, Casper van Eijck, Tom Luedde, Edgar Dahl, Jakob Nikolas Kather, Shivan Sivakumar, Wolfram Trudo Knoefel, Georg Wiltberger, Ulf Peter Neumann, Lara R. Heij

**Affiliations:** 1 Department of Surgery and Transplantation, University Hospital RWTH Aachen, Pauwelsstrasse 30, 52074 Aachen, Germany; 2 Research Institute of Pancreatic Diseases, Shanghai Key Laboratory of Translational Research for Pancreatic Neoplasms, Shanghai Jiao Tong University School of Medicine, Shanghai, China; 3 Department of General, Visceral and Transplantation Surgery, Universitats Klinikum Essen, Essen, Germany; 4 Department of Surgery, Maastricht University Medical Center, Maastricht, The Netherlands; 5 Department of Internal Medicine, Division of Medical Oncology, GROW, Maastricht University Medical Center, Maastricht, The Netherlands; 6 Department of Surgery, Erasmus University Medical Centre, Rotterdam, The Netherlands; 7 Department of Pathology, Erasmus University Medical Center, Rotterdam, The Netherlands; 8 Department of Gastroenterology, Hepatology and Infectious Diseases, University Hospital Duesseldorf, Duesseldorf, Germany; 9 Institute of Pathology, University Hospital RWTH Aachen, Aachen, Germany; 10 Else Kroener Fresenius Center for Digital Health, Medical Faculty Carl Gustav Carus, Technical University Dresden, Dresden, Germany; 11 Department of Immunology and Immunotherapy, School of Infection, Inflammation and Immunology, College of Medicine and Health, University of Birmingham, Birmingham, UK; 12 Department of General, Visceral and Pediatric Surgery, Heinrich Heine University, Düsseldorf, Germany; 13 Department of Surgery, Maastricht University Medical Center, Maastricht, The Netherlands; 14 Institute of Experimental Medicine and Systems Biology, RWTH Aachen University, Aachen, Germany; 15 Institute of Pathology, University Hospital Essen, Essen, Germany

**Keywords:** Pancreatic cancer, Prognostic biomarker, Deep learning, Tumor proportion, Immune infiltration, Oncogenes, Predictive markers, Prognostic markers

## Abstract

Pancreatic ductal adenocarcinoma (PDAC) is one of the most lethal cancers known to humans. However, not all patients fare equally poor survival, and a minority of patients even survives advanced disease for months or years. Thus, there is a clinical need to search corresponding prognostic biomarkers which forecast survival on an individual basis. To dig more information and identify potential biomarkers from PDAC pathological slides, we trained a deep learning (DL) model based U-net-shaped backbone. This DL model can automatically detect tumor, stroma and lymphocytes on whole slide images (WSIs) of PDAC patients. We performed an analysis of 800 PDAC scans, categorizing stroma in percentage (SIP) and lymphocytes in percentage (LIP) into two and three categories, respectively. The presented model achieved remarkable accuracy results with a total accuracy of 94.72%, a mean intersection of union rate of 78.66%, and a mean dice coefficient of 87.74%. Survival analysis revealed that SIP-mediate and LIP-high groups correlated with enhanced median overall survival (OS) across all cohorts. These findings underscore the potential of SIP and LIP as prognostic biomarkers for PDAC and highlight the utility of DL as a tool for PDAC biomarkers detecting on WSIs.

**Supplementary Information:**

The online version contains supplementary material available at 10.1038/s41598-025-94362-x.

## Background

PDAC is one of the most aggressive cancers with a high mortality rate. Currently, surgical therapy offers the only curative option for patients diagnosed at an early stage and adjuvant chemotherapy is essential to improve survival rates.

Biologically, PDAC is characterized by an abundant desmoplastic stroma, housing cancer-associated fibroblasts (CAFs), tumor vasculature and an extracellular matrix (ECM)^1^. This stromal compartment is thought to be held responsible for the poor efficacy of chemotherapy^1^. Clinically, pathologists use routine tissue sections stained by haematoxylin and eosin (H&E) to guide them in the tumor, node, and metastasis (TNM) staging.

The desmoplastic stroma is part of the tumor microenvironment (TME). Different stromal phenotypes exist in PDAC but are not comprehensively characterized. Emerging evidence shows that tumor-stroma ratio (TSR), gauged through either tumor in percentage (TIP) or stroma in percentage (SIP), as well as lymphocytes in percentage (LIP), has independent prognostic value for PDAC patients^2–7^. However, corresponding studies with large cohorts are scarce while results of small-scale studies have yielded inconclusive results.

Traditional TSR assessment relies on trained pathologists manual scoring or visually estimating tumor epithelial cells on H&E-stained sections, this method is ripe for enhancement through automated evaluation methods, yet, today, these automated methods remain immature and still cannot adequately replace for pathologists’ assessment in clinical application. The transition from traditional pathology to digital pathology, facilitated by advancements in imaging technologies and computational capabilities, has opened new avenues for more objective TSR evaluation^8^. Pathologists increasingly use digital slides to objectively evaluate pathological information. They manually outline tumor regions and analyze them on software platforms, such as QuPath^9^.

Although H&E imaging remains the gold standard for evaluation of PDAC diagnosis, there is a growing emphasis on leveraging high-dimensional imaging techniques. These techniques including imaging labeled tissue sections, multiplex immunohistochemistry, and imaging mass spectrometry enable simultaneous phenotyping of multiple cell populations, facilitating biomarker discovery within the TME. They have enabled deeper insights into the TME by providing spatial and molecular information far beyond what is possible with traditional H&E staining^10^. This shift toward high-dimensional imaging underscores its potential to complement and enhance routine pathology in biomarker identification and prognostic assessment^11^.

A pivotal aspect of digital pathology is DL, which has revolutionized the predictive modeling of diseases using image features. DL, particularly convolutional neural networks (CNNs), has performed in medical image segmentation, notably for simple organs, such as the colon^12^ and breast^13^. In recent years, research efforts of medical images analysis have increasingly focused on the segmentation of pathologically affected complex organs, such as the pancreas. Among CNNs, the U-net model has achieved superior performance in image segmentation due to its skip-connection scheme. This scheme connects low- and high- level features extracted by the encode to the decode to achieve a better feature representation^14^. U-net-shaped structures plus skip-connections are still the mainstream in medical image segmentation models.

To explore the prognostic value of TSR in PDAC and the application of CNNs for TSR assessment on digital H&E-stained PDAC image analysis, we applied CNNs through an automated detection of tumor, stroma and lymphocytes on WSI from PDAC patients. Subsequently, we aimed to validate their prognostic value across diverse international cohorts.

## Methods

### Patient cohorts

**Fig. 1:**
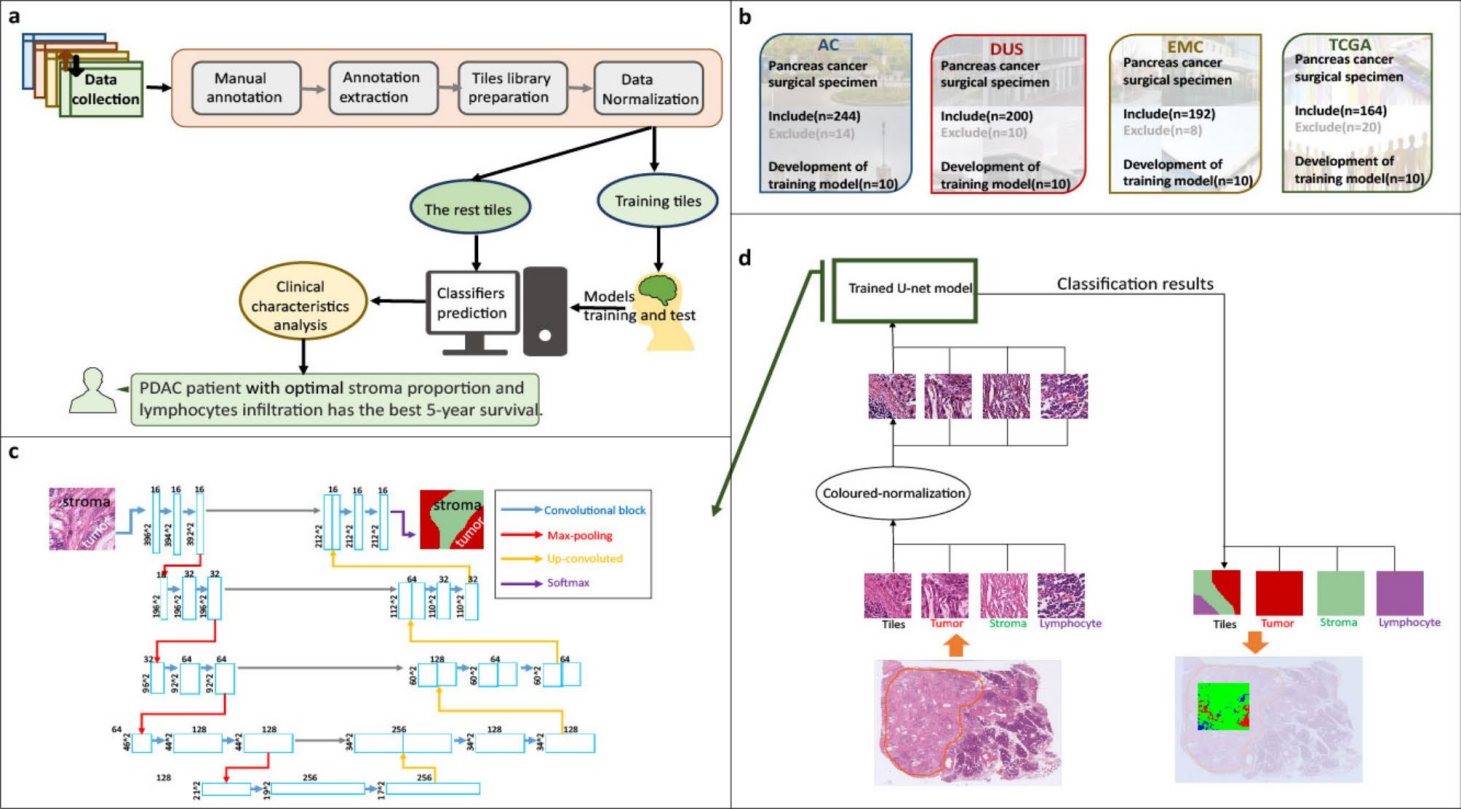
Study outline. (**a**) Overview of study workflow: after sample collection, images are processed using QuPath; then, models are trained and classification is performed, followed by data analysis. (**b**) Samples sources: the four cohorts (AC, DUS, EMC and TCGA) contributed histology slides and clinical data, all included patients underwent surgical resection for Pancreatic Ductal Adenocarcinoma. (**c**) The slide classification workflow: tiles from a whole digitized slide image are put into the trained U-Net classification model which is illustrated in (**d**). Output is a prediction map of aggregated tiles of the input whole slide. (d) Structure of used U-Net classification model: a U-Net architecture employed for image segmentation of 396 × 396 pixel patches. The image patch with its normalized RGB channels undergoes convolution and maxpooling iterations for feature extraction. The resulting feature maps are then up convoluted and concatenated with copies of the feature maps from the contracting path to retain location information four times. 10% of pixels are dropped between convolutions, which use the ReLU activation function with a 3 × 3 kernel. In the final step, the feature maps are convoluted with a 1 × 1 filter and a softmax activation function to create masks for the input image patch.

In this retrospective, multicenter cohort study, we included 800 histology slides from surgically resected PDAC patients from four sites (Fig. 1). The cohorts were named as followes AC from University Hospital Aachen Germany. DUS from the University Hospital Düsseldorf Germany. EMC from Erasmus Medical Center Rotterdam, the Netherlands. The Cancer Genome Atlas (TCGA) diagnostic slides were downloaded from the public TCGA data portal at https://portal.gdc.cancer.gov^15,16^. Informed consent was obtained from all participants. This study was conducted in accordance with the requirements of the Institutional Review Board of the RWTH-Aachen University (EK 005/21), the Declaration of Helsinki and the International Ethical Guidelines for Biomedical Research Involving Human Subjects by the Council for International Organizations of Medical Sciences (CIOMS), and good clinical practice guidelines (ICH-GCP). This study complied with the “Transparent reporting of a multivariable prediction model for individual prognosis or diagnosis” (TRIPOD) statement^17^.

### Image processing

**Fig. 2:**
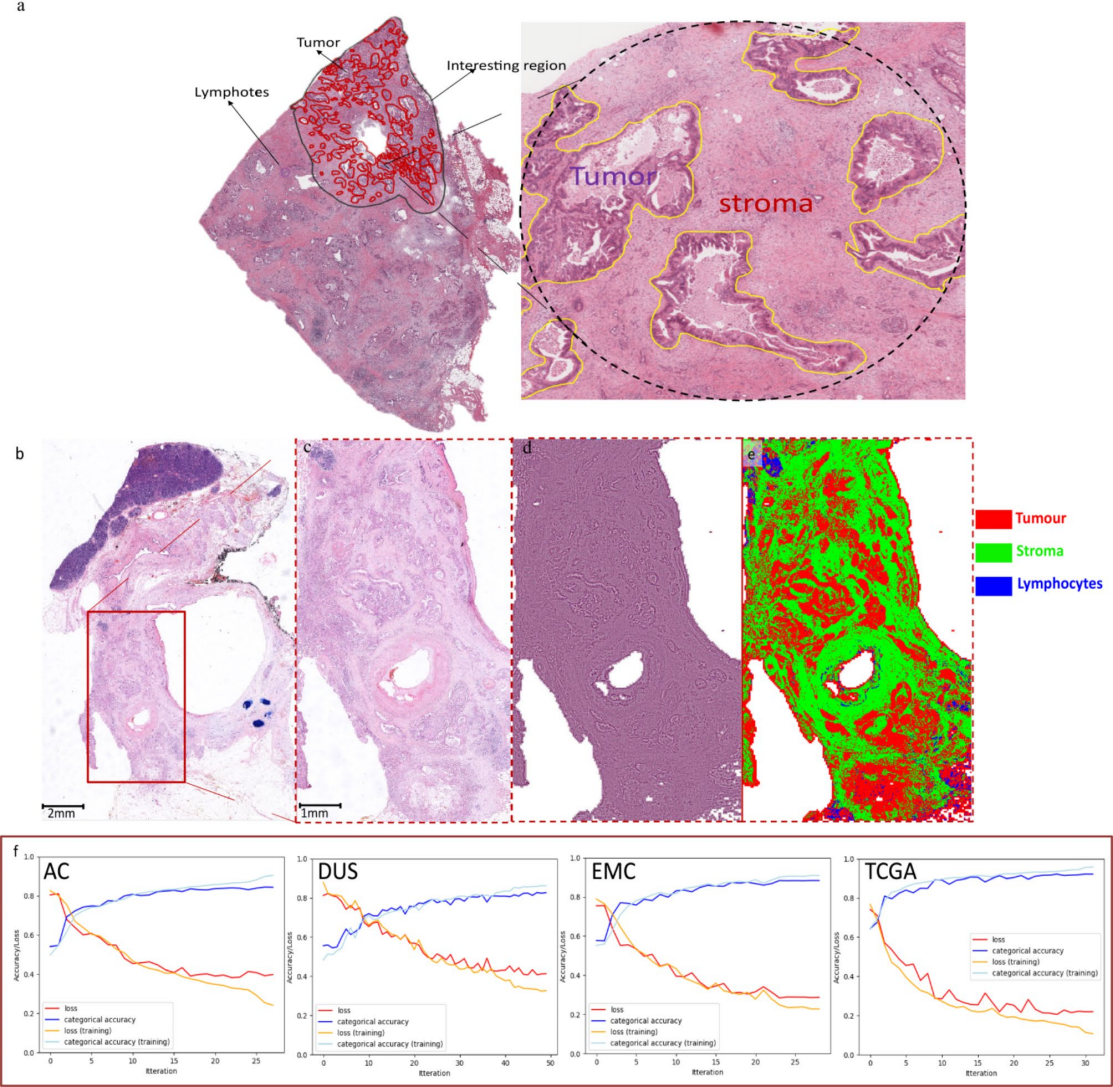
Classification map and output results of the U-net model. (**a**) A manual example from pathologists (**b**) a HE-stained WSI from AC cohort as an example, red box is circled as ROI which includes tumor, stroma, and lymphocytes; (**c**) the original extraction region from WSI; (**d**) the colored normalized ROI; (**e**) the classification map which includes tumor (red), stroma (green), and lymphocytes (blue). (**f**) loss and categorical accuracies of the four trained models. HE, hematoxylin and eosin.

Histological slides were selected by the pathologists and scanned at each institution. Each slide was manually outlined in a region of interest (ROI) including tumor glands, lymphocytes and desmoplastic stroma by senior pathologists using QuPath v0.2.4 (University of Edinburgh, Scotland)^9^. A manual reviewed example by pathologists is shown in Fig. 2a. In addition, we randomly selected 10 WSIs from each cohort, which were meticulously annotated by experienced pathologists to delineate stroma, lymphocytes, and tumor regions. From these annotated regions, we extracted over 50,000 representative image tiles for each tissue type per cohort, ensuring a comprehensive and balanced training set for model development. as training set from each cohort. The stromal area was defined by the following formula: $\:{A}_{Stroma}={A}_{ROI}-\left({A}_{Tumor}+{A}_{Lympℎocytes}\right)$. After annotating all of the training and verification slides, we tessellated the ROI into square image patches (tiles) using QuPath v0.2.4. The image patch size is 99 × 99 μm^2^, which consists of 396 pixels per side, and each pixel represents 0.25 μm of tissue at 20x magnification(396 pixels × 0.25 μm/pixel = 99 μm). This process generated a training image set containing over 50,000 image patches for each cohort.The training ROIs were extracted with a ternary mask, distinguishing between tumor, lymphocytes, and stroma regions. In total, over 50,000 training image patches were used to train a deep learning model based on a modified U-Net architecture. The input layer of the model had a size of 396 × 396 × 3 pixels, and the output layer had three categories: tumor, stroma, and lymphocytes. To account for potential color variation across sources, we applied color normalization to all tiles using the Macenko method^18^. Representative normalized images from each of the four sources are provided in the supplementary Fig. 1.

### Deep learning algorithm

We utilized a U-net as the backbone for segmentation learning because of its perfect performance in medical imaging segmentation^19^. The encoder of the U-net was realized by convolutional layers, followed by a max pooling operation. The bottleneck consisted of convolutions only, while the decoder used transpose convolutional layers. For all layers except the last, the ReLU activation layer was used. Because the predicted classes were exclusive, the softmax activation function was used in the final layers. Between the encoder and decoder layer, skip connections were used. The exact parameters and topology can be found in Fig. 1d. The U-net architecture was implemented in Python using the Keras library https://git.rwth-aachen.de/workgroup-lara/multiclass_segmentation_pancreatic_cancer. The project was run on a desktop computer with two INTEL(R) Xeon(R) Gold 6226R CPUs @ 2.90 GHz, two NVIDIA Quadro RTX 6000 GPUs and 128GB DDR4 RAM. Pixels lost during convolution were compensated by applying an overlapping tile strategy. The input dimensions were 396 × 396 × 3 while the output tile size was 212 × 212 × 3, as shown in Fig. 1. To prevent overfitting, between each convolution 10% of pixels were randomly dropped after each convolution. For convolution, a standard kernel size of a 3 × 3 convolution filter was chosen. The output of the classification contained a mask with three numerical classes representing stroma, tumor and lymphocytes. Hyperparameters were adjusted empirically. For the training, a batch size of 160 was used, when a plateau was reached in three consecutive training steps, the learning rate was reduced by 90%. Training was performed for up to 50 epochs or until the loss did not decrease for at least 5 training steps. The mean intersection over union, and average categorical accuracy was used to evaluate the performance of model.

### Statistical analysis

The potential cut-off of the stroma proportion among stroma-high, stroma-intermediate and stroma-low patients, associated with OS, was determined using X-tile^20^. The optimal cut-off to categorize lymphocyte proportion into lymphocytes-low and lymphocytes-high was determined using the maximally selected rank statistics method, a statistical approach utilized for selecting the optimal cut-off points when dividing a continuous variable into two categories. This method indicates the maximum statistical significance of the association between the continuous variable and the binary outcome^21^. Kaplan–Meier survival analysis was applied for the analysis of the survival curves, and log-rank test was used to test the differences in survival distribution. Group comparisons were conducted by the Mann-Whitney U test for continuous variables, while the χ^2^ test or linear-by-linear association in accordance with scale and number count were used in case of categorical variables.

All statistical analyses were performed in R unless otherwise noted (R version 3.6.4) using the following R packages: “survival”, “survminer”, and “psych”. A *p* < 0.05 was considered statistically significant. Neural network training and deployment were performed in Python (Python version 3.9.5).

## Results

### Patient characteristics

Patient characteristics grouped by stroma and lymphocytes proportion are summarized in Supplementary Table 1. Cases with perioperative mortality, defined as postoperative death within 30 days after surgery, were excluded from the analysis as stated. Further demographic and clinic pathological details of the cohort are outlined in Supplementary Table 1.

### Training model assessment

For each cohort, we trained and applied a DL model which was able to correctly distinguish tumor, stroma, and lymphocytes within the ROI. A classification map and output result of the U-net models are presented in Fig. 2: A magnified region (Fig. 2c) from a WSI (Fig. 2b) is processed using color normalization (Fig. 2d) and subsequently detected by a DL model (Fig. 2e). Supplementary Table 2 shows a summary of different evaluation metrics of the 4 cohorts on their test sets. Figure 2f shows a classification map along with the accuracy and loss of each cohort.

### Evaluation of an independent prognostic value of stroma in percentage

To assess the prognostic value of the predicted SIP, we analyzed a histogram for SIP individually based on the X-tile tools: X-tile plots were created by dividing SIP into three populations: SIP-low, SIP-intermediate, and SIP-high. Association was calculated at a three-group division by the log-rank test for survival in each cohort. The relationship between SIP predicted by the U-Net model and median OS showed two distinct subpopulations, SIP-high and SIP-low, both of which demonstrated a poor median OS rather than SIP-intermediate (supplementary Fig. 2). The optimal median OS cut-off point for SIP-intermediate of 4 cohorts was: AC (53.54-75.56%), DUS (53.79-70.87%), EMC (63.09-74.78%), TCGA (53.39%-72,20%). Based on the results above, we grouped SIP-low and SIP-high as SIP-LH (which combines combined SIP-high and SIP-low), hence patients were classified into two groups, SIP-intermediate and SIP-LH, for OS survival analysis.

**Fig. 3:**
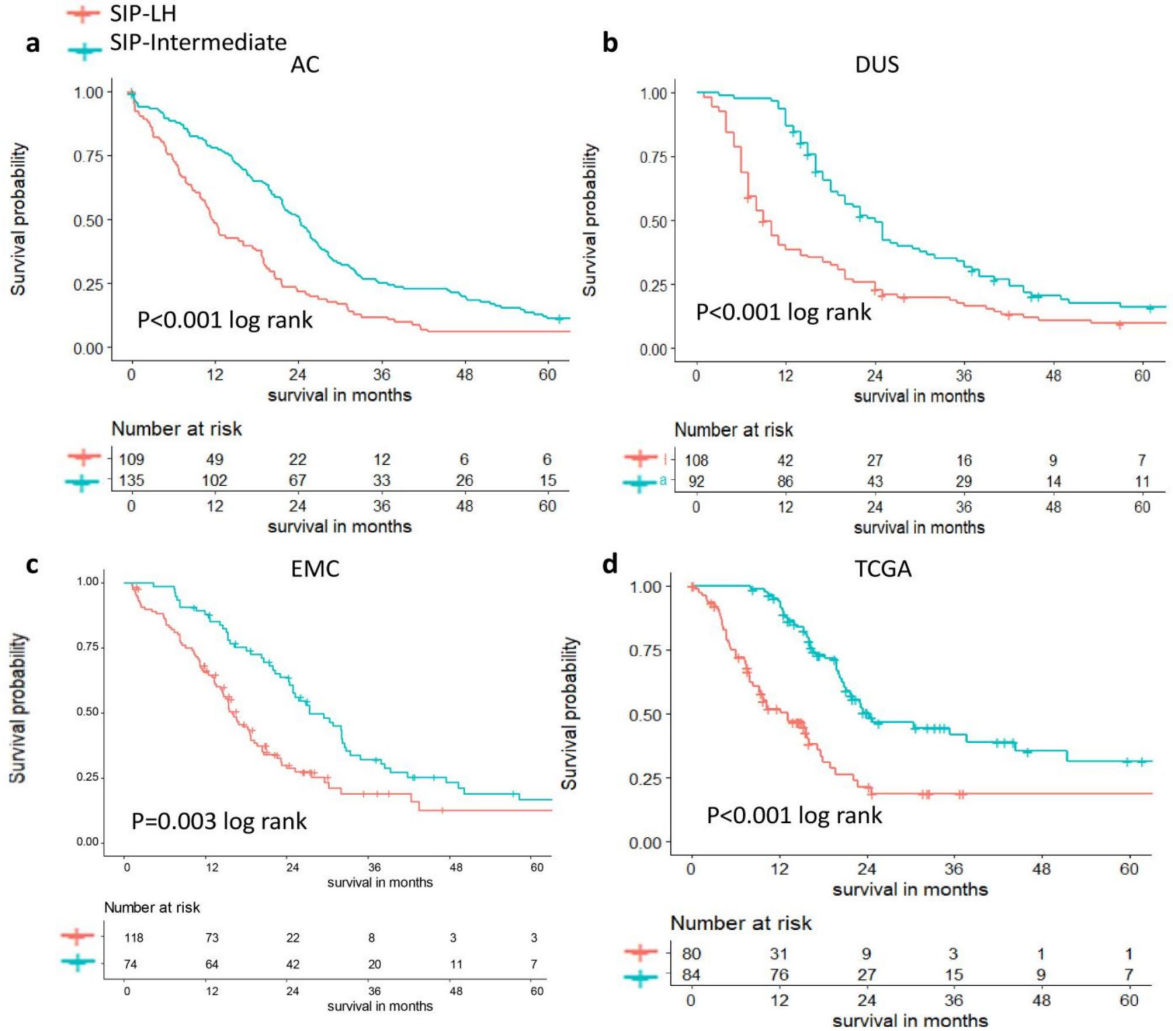
Oncological median OS in PDAC stratified by stroma in percentage (SIP). (**a**) The median OS of the AC cohort: the median OS was 24 months (95% CI: 22–27) in the SIP-intermediate group and 12 months (95% CI: 10–17) in the SIP-LH group; (**b**) median OS of the DUS cohort: the median OS was 24 months (95% CI: 20–30) in the SIP-intermediate group and 9 months (95% CI: 8–12) in the SIP-LH group; (**c**) 5- year OS of the EMC cohort: the median OS was 27 months (95% CI: 25–33) in the SIP-intermediate group and 16 months (95% CI: 15–19) in the SIP-LH group; (**d**) median OS of the TCGA cohort: the median OS was 24 months (95% CI: 21–51) in the SIP-intermediate group and 13 months (95% CI: 9–18) in the SIPLH group. SIP, stroma in percentage; OS, overall survival; SIP-LH, SIP-Low and SIP-High.

For AC, SIP-intermediate (*n* = 135) and SIP-LH (*n* = 109) demonstrated a median OS of 24 months (95%CI: 22–27) and 12 months (95%CI: 10–17) respectively, with log-rank test *p* < 0.001. For DUS, SIP-intermediate (*n* = 92) and SIP-LH (*n* = 108) median OS demonstrated 24 months (95%CI: 20–30) and 9 months (95%CI: 8–12) respectively, with log-rank test *p* < 0.001. For EMC, SIP-intermediate (*n* = 74) and SIP-LH (*n* = 118) median OS demonstrated 27 months (95%CI 25–33 and 16.0 months (95%CI: 15–19) respectively, with log-rank test *p* < 0.001. For TCGA, SIP-intermediate (*n* = 84) and SIP-LH (*n* = 80) median OS demonstrated 24 months (95%CI: 21–51) and 13 months (95%CI: 9–18) months respectively, with log-rank test *p* < 0.001 (Fig. 3).

### Evaluation of an independent prognostic value of lymphocytes in percentage

**Fig. 4:**
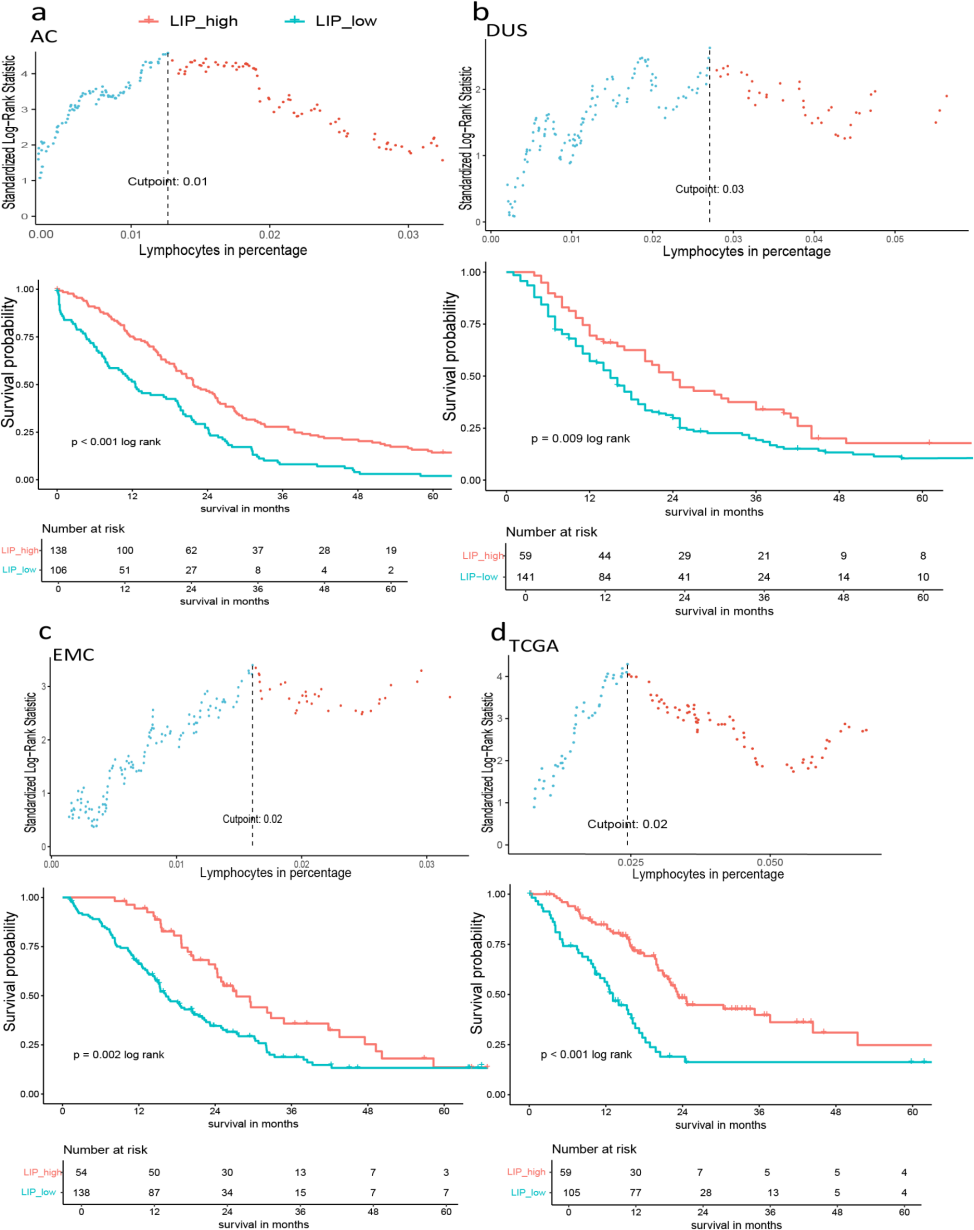
Optimal cut-off determinant of lymphocytes in percentage (LIP) and oncological median OS in PDAC stratified by LIP. (**a**) In AC, the optimal cut-off of LIP was 0.01, the median OS was 22 months (95% CI: 19–26) in LIP-high compared to 13 months (95% CI: 8–19) in LIP-low; (**b**) in DUS, the optimal cut-off of LIP was 0.03, the median OS was 24 months (95% CI: 20–36) in LIP-high compared to 15 months (95% CI: 12–18) in LIP-low; (**c**) in EMC, the optimal cut-off of LIP was 0.02, the median OS was 27 months (95% CI: 24–43) in LIP-high compared to 16 months (95% CI: 15–21) in LIP-low; (**d**) in TCGA,, the optimal cut-off of LIP was 0.02, the median OS was 23 months (95% CI: 21–51) in LIP-high compared to 13 months (95% CI: 10–17) in LIP-low. LIP, lymphocytes in percentage; OS, overall survival.

A cut-off value for LIP was determined by an optimal cut-off point which classified LIP into LIP-low and LIP-high. Figure 4 presents the optimal cut-off for LIP in each cohort. A comparative group analysis regarding lymphocytes and median OS was carried out between patients with LIP-low and LIP-high. Kaplan-Meier survival analysis demonstrated high lymphocyte infiltration to have a longer median OS: AC, the median OS demonstrated 22 months (95%CI: 19–26) in LIP-high (*n* = 138) compared to 13 months (95%CI: 8–19) in LIP-low (*n* = 106)(*p* < 0.001 log rank); For DUS, 24 months (95%CI: 20–36) in LIP-high (*n* = 59) compared to 15 months (95%CI: 12–18) in LIP-low (*n* = 141) (*p* = 0.009 log rank); For EMC, 27 months (95%CI: 24–44) in LIP-high (*n* = 54) compared to 16 months (95%CI: 15–21) in LIP-low (*n* = 138) (*p* = 0.002 log rank); For TCGA, 23 months (95%CI: 21–44) in LIP-high (*n* = 105) compared to 13 months (95%CI: 10–17) in LIP-low (*n* = 59) (*p* < 0.001 log rank).

### Evaluation of a combined prognostic value of stroma-in-percentage and lymphocytes-in-percentage

To summarize the results described above, SIP-intermediate and LIP-high demonstrated a better survival compared with SIP-LH and LIP-low. Further, to evaluate a combined effect of stroma and lymphocytes, we analyzed the median OS with the combination of SIP and LIP, thus, we classified each cohort into 3 groups:

Group 1: SIP-intermediate and LIP-high- with both optimal stroma and high lymphocytes infiltration.

Group 2: SIP-intermediate and LIP-low or SIP-LH and LIP-high: either optimal stroma in percentage or high lymphocytes infiltration.

Group 3: SIP-LH and LIP-low- both classes with poor prognostic value for stroma in percentage and low lymphocytes.

**Fig. 5:**
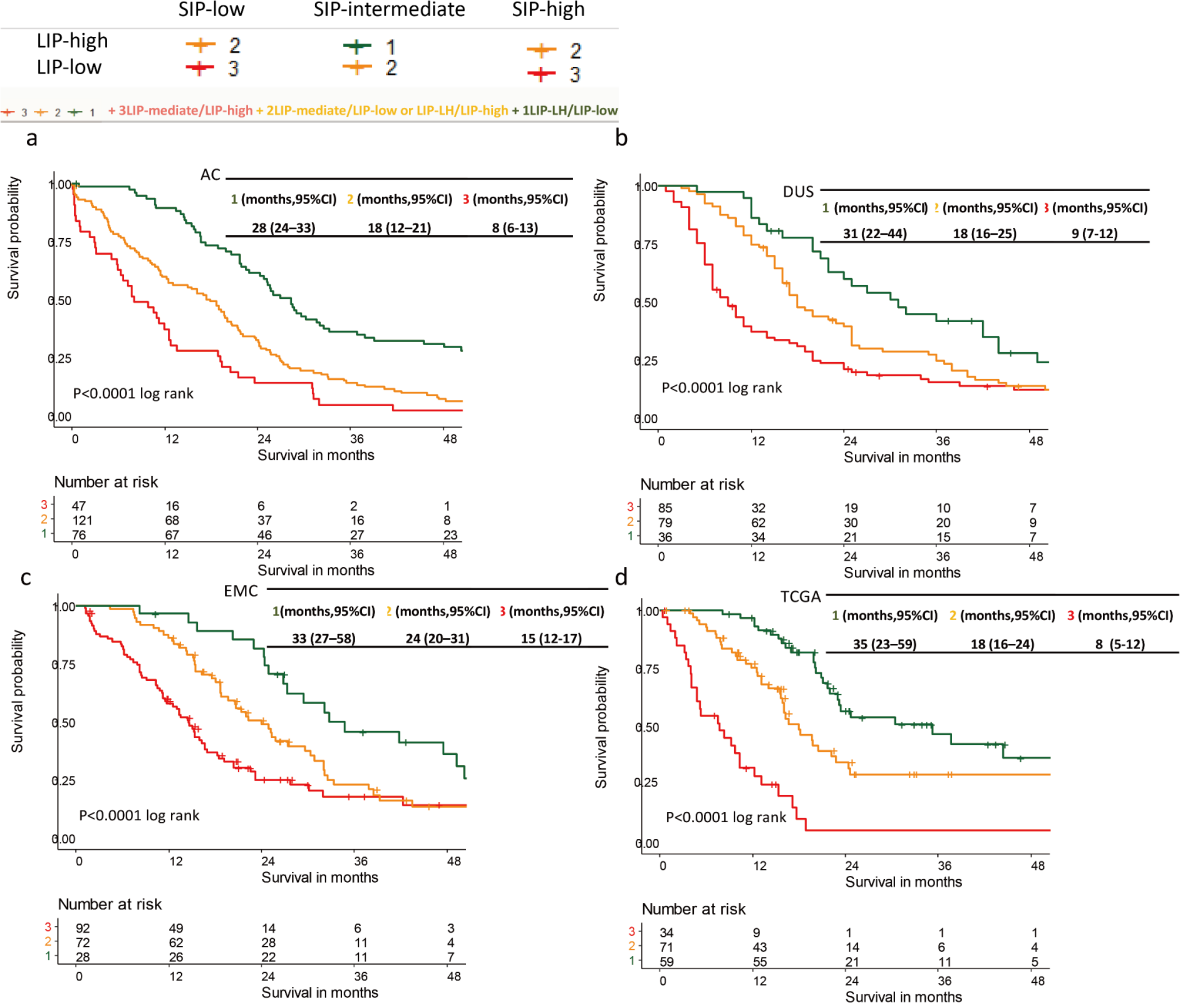
Oncological survival in PDAC analyzed by combination of SIP and LIP. Group 1, whose patients are both SIP-intermediate and LIP-high, had the best median OS. (**a**) In AC, there was a median OS of 28 months (95%CI: 24–33) in group 1; (**b**) in DUS, there was a median OS of 31 months (95%CI: 22–44) in group 1; (**c**) in EMC, there was a median OS of 33 months (95%CI: 27–58) in group 1; (**d**) in TCGA, there was a median OS of 35 months (95%CI: 23–59) in group 1.

The results of a subsequent Kaplan-Meier analysis are presented in Table 1; Fig. 5. Group 1 with the combination of SIP-intermediate and LIP-high had the best median OS:

AC demonstrated a median OS of 28 months (95%CI: 24–33) in group 1 (*n* = 76), group 1 v.s other groups HR 0.44 (0.32–0.59), *p* < 0.001, 18 months (95%CI: 12–21) in group 2 (*n* = 121), group 1 VS group 2, HR = 0.29, (95%CI 0.19–0.43), and 8 months (95%CI: 6–13) in group 3 (*n* = 47) (*p* < 0.001 log rank), group 1 VS group 3, HR = 0.59, (95%CI 0.41–0.84). For DUS, the combination of SIP-intermediate and LIP-high resulted in a median OS of 31 months (95%CI: 22–44) in group 1 (*n* = 36), group 1 v.s other groups HR 0.49 (0.32–0.75), *p* = 0.001, 18 months (95%CI: 16–25) in group 2 (*n* = 79), group 1 VS group 2, HR = 0.59, (95%CI 0.43–0.82), and 9 months (95%CI: 7–12) in group 3 (*n* = 85) (*p* < 0.001 log rank), group 1 VS group 3, HR = 0.31, (95%CI 0.21–0.47). For EMC, the combination of SIP-intermediate and LIP-high resulted in a median OS of 33 months (95%CI: 27–58) in group 1 (*n* = 28), group 1 v.s other groups HR 0.46 (0.28–0.76), *p* = 0.002, 24 months (95%CI: 20–31) in group 2 (*n* = 72), group 1 VS group 2, HR = 0.57, (95%CI 0.39–0.83), and 15 months (95%CI: 12–17) in group 3 (*n* = 92) (*p* < 0.001 log rank), group 1 VS group 3, HR = 0.35, (95%CI 0.21–0.58). For TCGA, the combination of SIP-intermediate and LIP-high resulted in a median OS of 35 months (95%CI: 23–59) in group 1 (*n* = 59), group 1 v.s other groups HR 0.37 (0.23–0.58), *p* = 0.002, 18 months (95%CI: 16–24) in group 2 (*n* = 71)), group 1 VS group 2, HR = 0.37, (95%CI 0.23–0.58), and 8 months (95%CI: 5–12) in group 3 (*n* = 34) (*p* < 0.001 log rank), group 1 VS group 3, HR = 0.13, (95%CI 0.07–0.24).

**Table 1:** Results of a subsequent Kaplan-Meier analysis.

	5 years OS (months)			HR(95%CI)		
Study Cohorts	Group 1	Group 2	Group 3	Group 1 VS Group 2	Group 1 VS Group 3	Group 1 VS Others*
AC	28 (24–33)	18 (12–21)	8 (6–13)	0.29 (0.19–0.43)	0.59 (0.41–0.84)	0.44 (0.32–0.59)
DUS	31 (22–44)	18 (16–25)	9 (7–12)	0.59 (0.43–0.82)	0.31 (0.21–0.47)	0.49 (0.32–0.75)
EMC	33 (27–58)	24 (20–31)	15 (12–17)	0.57 (0.39–0.83)	0.35 (0.21–0.58)	0.46 (0.28–0.76)
TCGA	35 (23–59)	18 (16–24)	8 (5–12)	0.37 (0.23–0.58)	0.13 (0.07–0.24)	0.37 (0.23–0.58)

*Others: a combination of group 2 and group 3

### Cox regression analysis

The univariate analysis is shown in Supplementary Table 3, all variables with a *p*-value < 0.05 were included in a multivariate Cox regression model. Here, AC demonstrated SIP (HR = 0.53, *p* < 0.001) and LIP (HR = 0.52, *p* < 0.001) as independent predictors of median OS. For DUS, the multivariate Cox regression model, SIP (HR = 0.53, *p* < 0.001) was identified as an independent predictor of median OS. For EMC,, SIP (HR = 0.68, *p* = 0.04) and LIP (HR = 0.65, *p* = 0.04) were identified as independent predictors. For TCGA, the multivariate Cox regression model, SIP (HR = 0.3, *p* < 0.001) and LIP (HR = 0.33, *p* < 0.001) were identified as independent predictors of median OS.

## Discussion

**Fig. 6:**
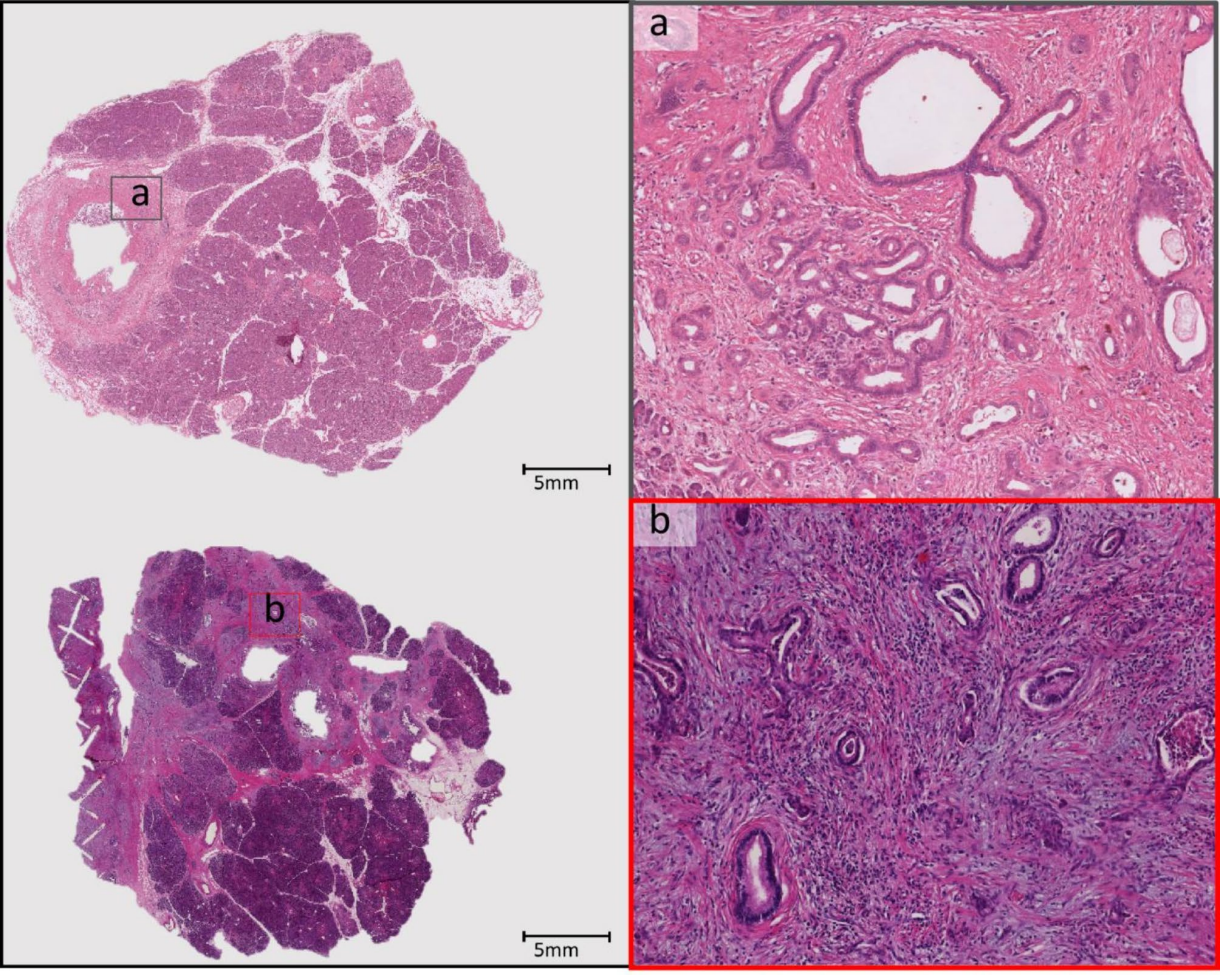
Representative H&E-stained sections of normal pancreas tissue and PDAC. (**a**) Shows a normal gland, while Panel (**b**) depicts a tumor gland.

The effects of the stroma proportion on oncological outcome have been inconsistent in previous studies^3,4,22^. Some studies have mentioned that a low stromal proportion was associated with cancer metastasis and poor prognosis^3,4,22^, while others reported the median OS in patients with high stroma proportion was shorter than that in patients with a relatively lower stroma proportion^5,6^. Accurately identifying tumor glands in PDAC is challenging, even for experienced pathologists, due to the inherent tumor heterogeneity. Similarly, some deep learning models, such as QuPath’s pixel classifier, often misclassify normal glands as tumor glands because the shape of normal glandular structures can closely resemble tumor glands in PDAC. For example, normal glands (Fig. 6a) and tumor glands (Fig. 6b) exhibit similar morphological characteristics. Additionally, lymphocytes present a similar appearance to cell nuclei in terms of hue that may further complicate the classification.

**Fig. 7:**
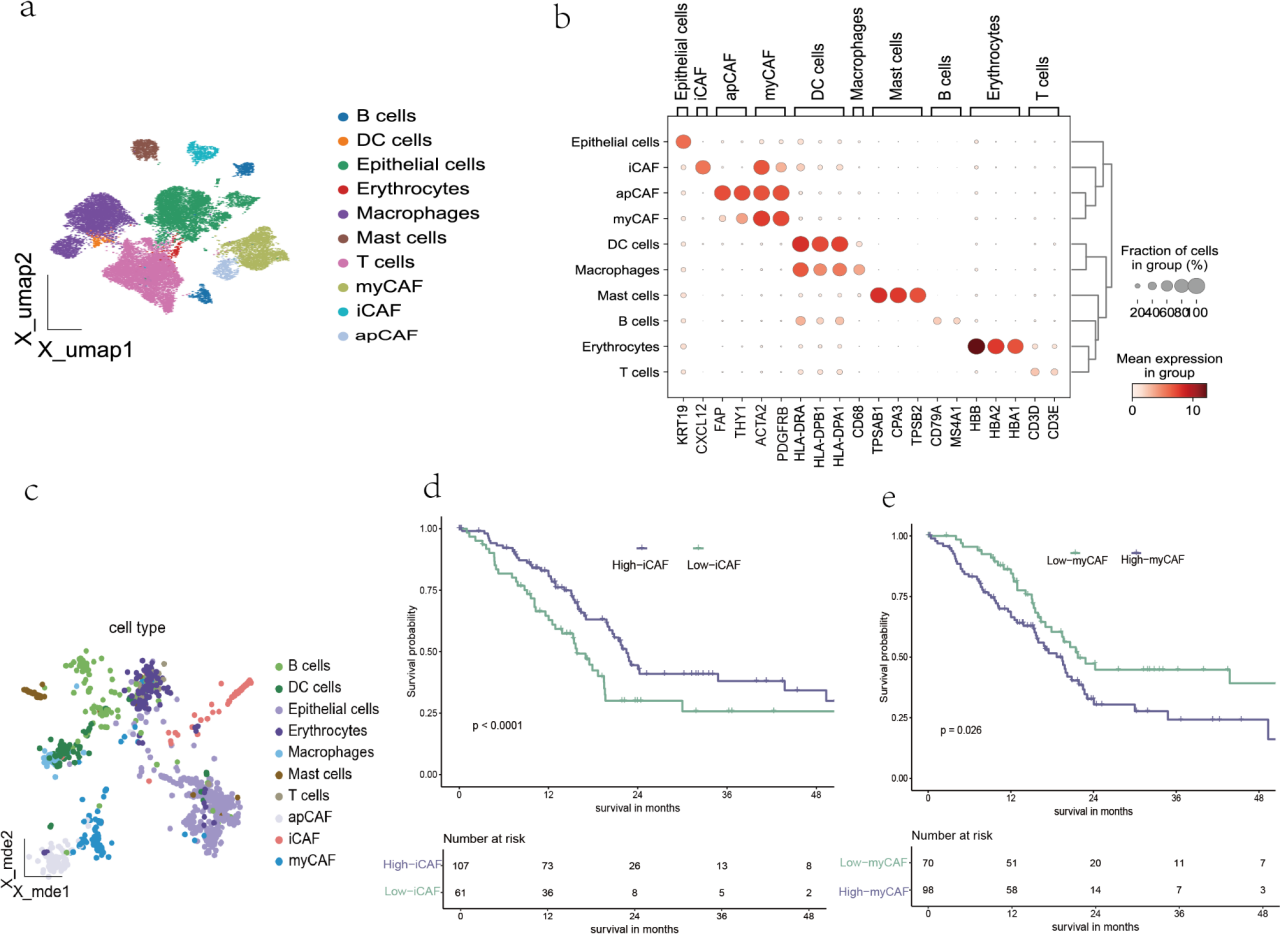
CAF subtypes are related with survival prognosis. (**a**) Umap plot showing 11 cell subsets of GSM6204111P03 data; (**b**) Dot plot showing key marker gene expression in each cell type of human primary PDAC tumor tissue; (**c**) Mde plot showing 11 cell subsets of TCGA cohort; (**d**) Kaplan-Meier curves showing the survival difference between High- and Low- abundance of iCAF; (**e**) Kaplan-Meier curves showing the survival difference between High- and Low- abundance of myCAF.

In our large study of 800 PDAC patients, we investigated the stromal and lymphocytes proportion with an automated image analysis approach. Using DL, the U-Net achieved an accuracy of 94.72% in the testing set of the TCGA cohort (Supplementary Table 2). Our results are reflective of the underlying biological heterogeneity in PDAC. Neither SIP-low nor SIP-high were associated with a better median OS. Increased tissue stiffness can activate pro-survival and pro-proliferation signaling pathways in cancer cells^23^. Additionally, enhanced mechanical stress may lead to the collapse of blood vessels, resulting in hypoxia, which promotes a shift toward more aggressive cancer phenotypes^24^. However, studies report that removing stroma completely resulted in more aggressive tumors with worse OS^25,26^. A possible underlying explanation for this paradoxical phenomenon is the heterogeneity of stroma. CAFs and pancreatic stellate cells (PSCs), are reported to create a tumor supportive microenvironment^27^. It has been reported that subtypes of CAFs with distinct phenotypes exist in PDAC.Single cell RNAseq identified inflammatory CAFs (iCAFs), myofibroblastic CAFs (myCAFs) and antigen-presenting CAFs (apCAFs) as functionally distinct subpopulations^28,29^. To assess the composition of CAF subtypes in the TCGA cohort, we obtained RNA expression data from https://portal.gdc.cancer.gov/projects/TCGA-PAAD, and used the primary PDAC single-cell RNA-seq data from GSM6204111P03 as a reference. We applied Bulk2Single deconvolution algorithm to convert Bulk RNA-seq data to Single Cell RNA-seq data^30^. Unsupervised clustering of human primary PDAC single-cell profiles identified 11 cell subsets(Fig. 7a), which we annotated by known gene signatures (Fig. 7b). Figure 7c shows cell profiles in TCGA cohort which is predicted by the Bulk2Single deconvolution algorithm. We assessed the prognostic difference between high or low abundance of CAF subtypes. Interestingly, the higher iCAF is related to a better prognosis(Fig. 7d), whereas higher myCAF is related to a worse one (log-rank *P* < 0.0001 and 0.026)(Fig. 7d). apCAF seems to have no prognostic impact. In addition, myCAFs exhibited juxtaposition with tumor cells, whereas iCAFs resided distally, potentially fostering an inflammatory environment^31^, which shows different ecotypes and infects the functions of stroma. The further mechanisms by which the CAF composition varies during cancer development remain unknown. Although the role of those CAF-subtypes within the TME is not fully understood yet, the different subtypes have been linked to either promote or block the tumor, revealing a complex role of CAFs in the TME^1,32^. Other components of the TME are known to facilitate the host in an anti-tumor response. These components potentially are small nerve fibers^33,34^, immune cells such as cytotoxic CD8 cells^35^ and lymphoid aggregates^36^. Also, the ECM may act as a physical barrier, limiting tumor invasion and metastasis^26,37^. Acquisition of a mesenchymal-like phenotype renders cancer cells into being more invasive and resistant to therapy-induced apoptosis^1,22,38^. However, the mechanisms behind these survival differences remain complex and warrant further investigation, particularly concerning stroma phonotypes and stromal-tumor interactions. To summarize, the stroma cell types present a high heterogeneity, which explains why patients with lower proportion of stroma also experience worse outcomes^39,40^.

The prognostic role of the proportion of lymphocytes in PDAC is extensively discussed^27,28^. Tumor infiltrating lymphocytes (TILs) are known to be associated with a better prognosis. In addition, a study presented a reduction of the total lymphocytes in blood samples as the main immunologic change in advanced PDAC patients and decreased total lymphocytes counts predicted a poor outcome^29^. In line with these previous observations, our results demonstrated that high proportions of TILs were associated with a better survival in patients with PDAC. Nevertheless, the presence of TILs is usually a good predictor for a better survival, but co-expression of checkpoint inhibitors influences their functional state.

Compared with prior studies, our approach offers some advancements. Traditional TSR assessment relies heavily on manual evaluation, which is subjective and labor-intensive. By employing CNNs, we demonstrated an automated, objective, and reproducible method for TSR evaluation across diverse international cohorts. This study builds on prior research by integrating deep learning with digital pathology to enhance the prognostic assessment of PDAC patients. While previous studies have shown the prognostic value of TSR, they have largely been limited by small cohort sizes. Our application of DL method allows for large-scale analysis, thereby improving the generalizability and robustness of TSR-based prognostication.

Demonstration of DL model as a viable tool for automated stroma and lymphocytes evaluation in PDAC, which bridges the gap between traditional pathology and high-dimensional computational approaches. In addition, we validated our applications across international cohorts.

However, there are limitations to consider. First, although our model achieved relatively high segmentation accuracy, its performance may be influenced by variations in image quality and slide preparation across institutions. This could introduce variability in cell populations assessment.

Second, while our model effectively distinguishes tumor, stroma, and lymphocytes, the accurate identification of other cell populations is planned for future studies. Additionally, the integration of our findings with other biomarkers and modalities, such as genetic or transcriptomic data, will be explored in further studies. A multimodal approach might provide a more comprehensive understanding of PDAC biology and improve risk stratification.

Lastly, while our study focused on survival analysis, functional studies can elucidate the biological mechanisms underlying the observed results.

## Conclusion

We presented a U-Net model to automatically determine tumor, stroma, and lymphocytes proportion using HE slides. The combination of SIP and LIP provides a risk stratification for PDAC patients undergoing surgery. Our study identified and provided further evidence for the prognostic value of stroma proportion for oncological outcome. Although our U-Net models are just the first of many steps in translating DL models into real clinical applications, our models indicate a promising start. We hope our study will provide valuable information to advance the application of DL in routine pathology.

## Electronic supplementary material

Below is the link to the electronic supplementary material.

Supplementary Material 1

## Data Availability

Due to NHIRD confidentiality, the linked data are not publicly available. The data will be provided upon reasonable request from Dr. Lara R. Heij: lararosaline.heij@uk-essen.de. The images and patient data for the TCGA cohort can be found at https://portal.gdc.cancer.gov.For cohorts from AC, DUS, and EMC, the images and patient data are from our collaborator. The source codes used for training and evaluating Deep Learning classifiers are publicly available at https://git.rwth-aachen.de/workgroup-lara/multiclass_segmentation_pancreatic_cancer.

## Abbreviations

AC: RWTH Aachen
apCAFs: Antigen-presenting CAF
CAF: Cancer-associated-fibroblast
CIOMS: Council for International Organizations of Medical Sciences
CNN: Convolutional neural network
DL: Deep learning
DUS: University Hospital Düsseldorf
ECM: Extracellular matrix
EMC: Erasmus Medical Center
FFPE: Formalin-fixed paraffin-embedded
HE: Hematoxylin and eosin
HR: Hazard ratio
iCAF: Inflammatory CAF
IL-1: The Interleukin-1 family
IL-6: The Interleukin-6 family
IOU: Intersection of Union
LIP: Lymphocytes in percentage
myCAF: Myofibroblastic CAF
OS: Overall survival
PDAC: Pancreatic ductal adenocarcinoma
ROI: Region of interest
SIP: Stroma in percent
TCGA: The cancer genome atlas
TGF- β: Transforming growth factor beta
TILs: Tumor infiltrating lymphocytes
TLS: Tertiary lymphoid structures
TME: Tumot microenvironment
TNFα: Tumor necrosis factor-alpha
TNM: Tumor, node and metastasis
TRIPOD: Transparent reporting of a multivariable prediction model for individual prognosis or diagnosis
TSR: Tumor-stroma ratio
WSI: Whole slide image
