# Supplementary material for: Stroma and lymphocytes identified by deep learning are independent predictors for survival in pancreatic cancer
Source: Sci Rep. 2025 Mar 19;15:9415. doi: 10.1038/s41598-025-94362-x (PMC11923104; doi:10.1038/s41598-025-94362-x)
Supplement: Supplementary file 1 — Supplementary Material 1 [file 41598_2025_94362_MOESM1_ESM.pdf]

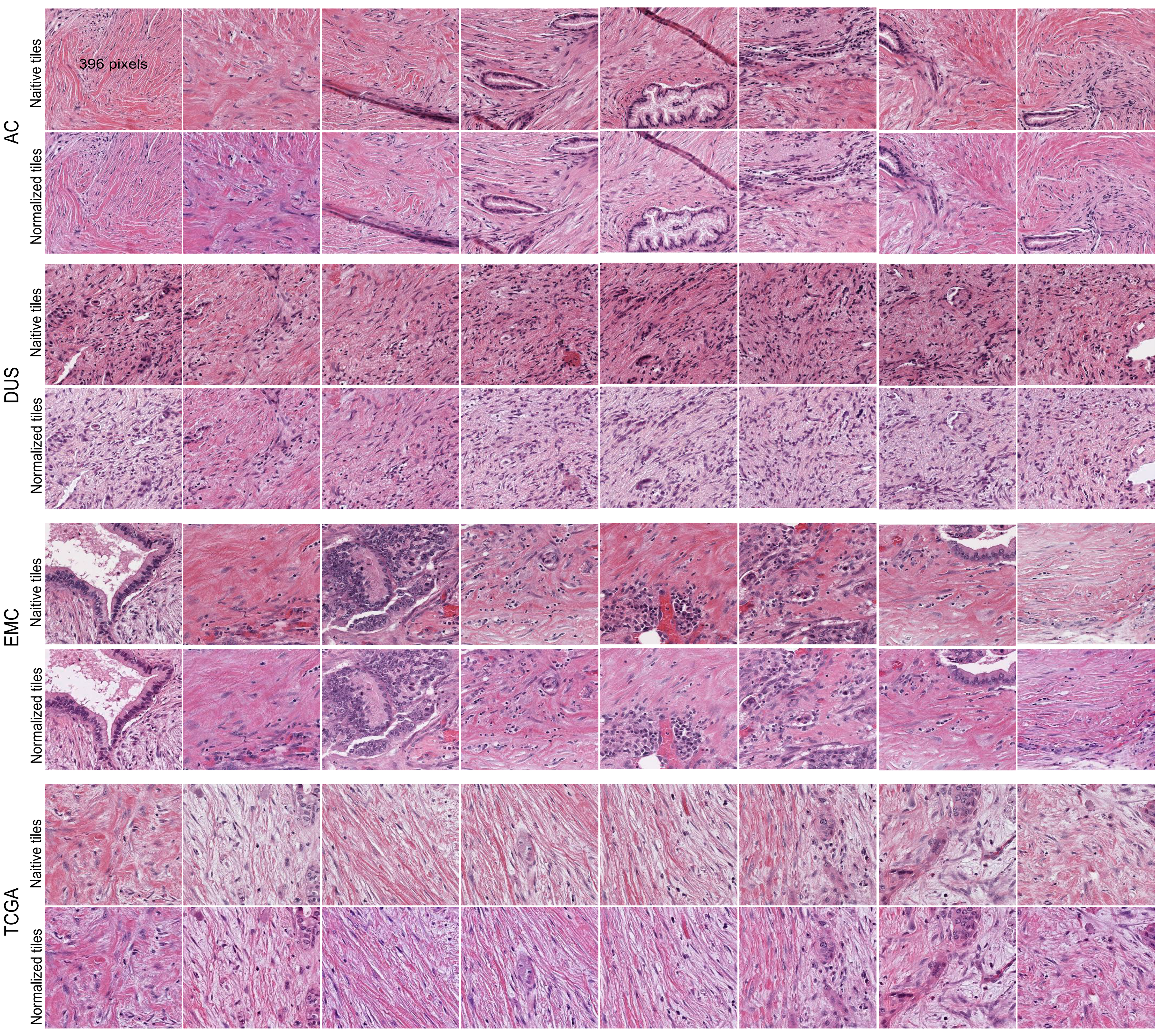

Supplementary Figure 1: Representative naïve tiles and corresponding normalized images from the four cohorts. Each tile is 396 × 396 pixels in size.

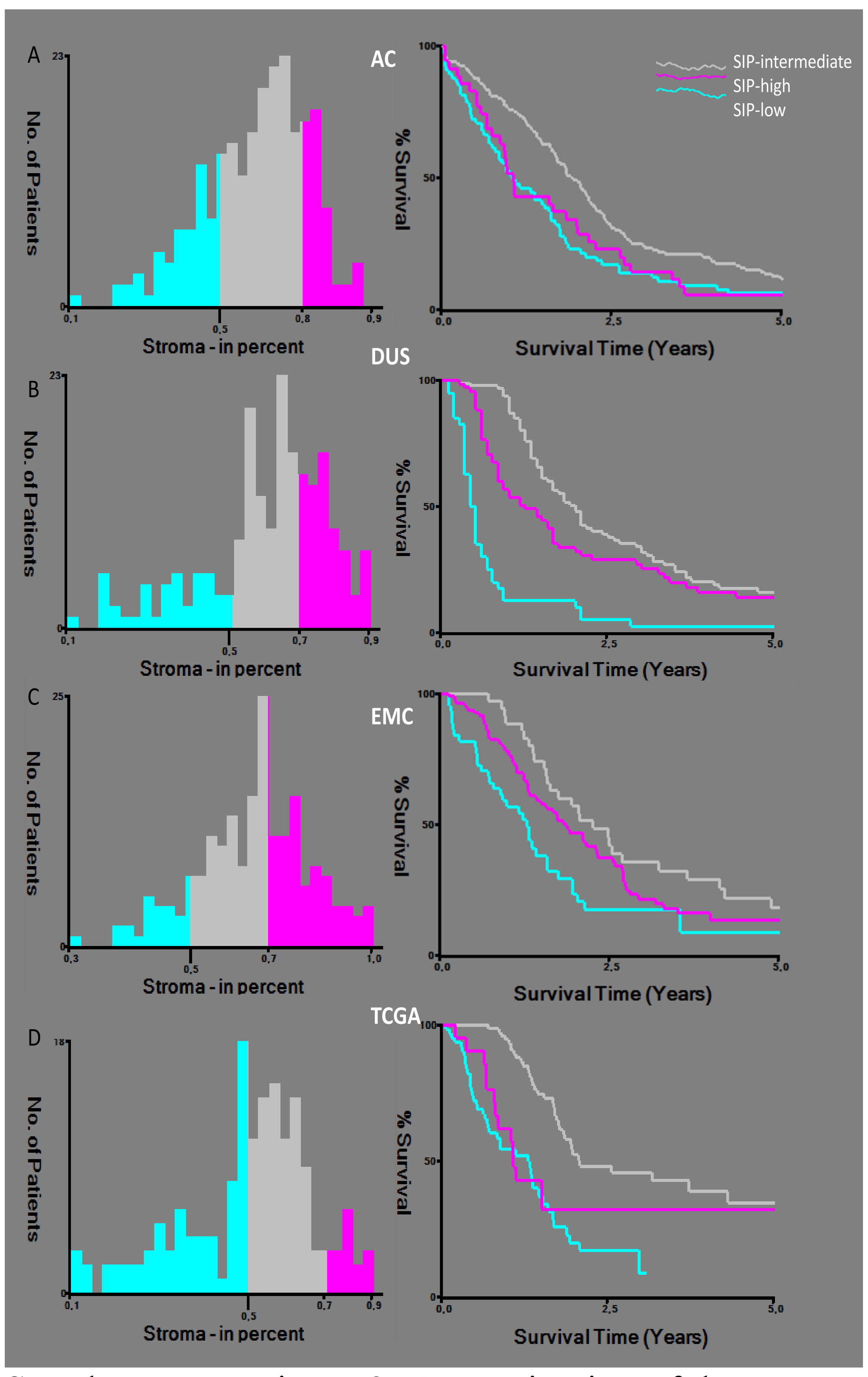

Supplementary Figure 2: Determination of the optimal cut-off for SIP using the X-tile tool (left) and corresponding overall survival in PDAC stratified by SIP (right) across the four cohorts.

Supplementary table 1 Patients' Characteristics

AC	SIP-intermediate (n=135)	SIP-LH (n=109)	p-value	LIP-low (n=106)	LIP-high (n=138)	p-value
Age: median (95%CI)	67 (36-86)	68.5 (48-84)	0,1198	767.5 (42-84)	68.5 (36-86)	0,4308
Gender	130	106	0,6169	102	134	0,2972
Male	63	47		52	76	
Female	67	59		50	58	
T-stage	n=128	n=106	0,5494	n=100	n=134	0,9604
T1	4	6		5	5	
T2	13	14		11	16	
T3	106	80		79	107	
T4	5	6		5	6	
N-stage	n=128	n=106	0,3261	n=100	n=134	0,1326
N0	29	33		33	29	
N1	88	66		59	95	
N2	11	7		8	10	
Lymphatic invasion	n=134	n=103	0,5521	n=102	n=135	0,3482
L0	93	76		69	100	
L1	41	27		33	35	
Vascular invasion	n=134	n=103	0,3674	n=102	n=135	0,0952
V0	115	83		80	118	
V1	19	20		22	17	
Perineural invasion	n=134	n=103	0,2875	n=102	n=135	0,1555
Pno	25	26		17	34	
Pn1	109	77		85	101	

DUS	SIP-intermediate (n=92)	SIP-LH (n=108)	p-value	LIP-low (n=141)	LIP-high (n=59)	p-value
T-stage	n=92	n=108	0,5119	n=141	n=59	0,4802
T1	1	0		0	1	
T2	2	5		5	2	
T3	86	98		130	54	
T4	3	5		6	2	
N-stage	n=92	n=107	0,3519	n=140	n=59	0,8653
N0	12	21		23	10	
N1	79	86		116	49	
N2	1	0		1	0	
Lymphatic invasion	n=88	n=55	0,8407	n=95	n=46	0,6635
L0	38	22		58	38	
L1	50	33		37	25	
Vascular invasion	n=87	n=54	1,0000	n=95	n=46	**0,0172**
V0	59	37		58	38	
V1	28	17		37	8	
Perineural invasion	n=62	n=31	0,9275	n=66	n=27	0,5772
Pno	12	7		12	7	
Pn1	50	24		54	20	
Grade	n=89	n=102	0,5356	n=133	n=58	0,8029
G0	0	1		1	0	
G2	48	50		68	30	
G3	41	51		64	28	

EMC	SIP-intermediate (n=74)	SIP-LH (n=118)	p-value	LIP-low (n=138)	LIP-high (n=54)	p-value
T-stage	n=74	n=118	0,1790	n=138	n=54	0,7582
T1	0	1		1	0	
T2	4	15		12	7	
T3	6	15		15	6	
T4	64	87		110	41	
N-stage	n=74	n=118	0,5220	n=138	n=54	0,9057
N0	21	40		43	18	
N1	53	78		95	36	
Lymphatic invasion	n=55	n=101	1,0000	n=113	n=43	0,5057
L0	20	37		39	18	
L1	35	64		74	25	
Perineural invasion	n=61	n=105	0,2823	n=118	n=48	0,4730
Pno	11	28		30	9	
Pn1	50	77		88	39	
Grade	n=69	n=102	0,3845	n=118	n=53	0,3055
G0	3	1		3	1	
G2	49	70		77	42	
G3	17	30	Supplementary	37	10	
G4	0	1		1	0	

TCGA	SIP-intermediate (n=84)	SIP-LH (n=80)	p-value	LIP-low (n=60)	LIP-high (n=104)	p-value
T-stage	n=83	n=80	0,9567	n=58	n=105	0,5415
T1	3	3		1	5	
T2	10	12		6	16	
T3	68	63		49	82	
T4	2	2		2	2	
N-stage	n=84	n=79	0,0544	n=58	n=105	0,0363
N0	27	14		9	32	
N1	54	64		46	72	
N2	3	1		3	1	

Age data are presented as mean and interquartile range. SIP. Stroma in percentage; LIP. Lymphocyte in percentage.

Supplementary table 2 Evaluation Metrics of the 4 Cohorts on the Test Set

Study cohorts	Total accuracy	Total mean error	Mean IOU rates	Mean dice coefficient Total
AC Cohort	0.8916	0.1084	0.6687	0.7985
TCGA Cohort	0.9472	0.0528	0.7866	0.8774
DUS Cohort	0.8882	0.1118	0.7257	0.8404
EMC Cohort	0.9243	0.0757	0.7551	0.8591
**Study cohorts**	**Specificity Total**	**Specificity Stroma**	**Specificity Tumor**	**Specificity Immune**
AC Cohort	0.8988	0.7977	0.9101	0.9884
TCGA Cohort	0.9470	0.9518	0.9000	0.9893
DUS Cohort	0.8884	0.7633	0.9047	0.9973
EMC Cohort	0.9242	0.9103	0.8642	0.9979
**Study cohorts**	**Sensitivity Total**	**Sensitivity Stroma**	**Sensitivity Tumor**	**Sensitivity Immune**
AC Cohort	0.8106	0.8937	0.7882	0.7500
TCGA Cohort	0.8859	0.8737	0.9502	0.8338
DUS Cohort	0.8236	0.8957	0.7554	0.8197
EMC Cohort	0.8396	0.8568	0.9131	0.7488

Supplementary table 3 Supplementary table 3. Univariate and Multivariate Analysis of the 5-Year OS in PDAC

	Univariate analysis		Multivariate analysis	
	HR(95%)	p value	HR(95%)	p value
AC				
T-stage(T1/T2=1)	0.98 (0.73-1.3)	0.88		
N-stage(N0=1)	1.1 (0.8-1.4)	0.71		
Stage(stage1=1)	1.1 (0.86-1.5)	0.36		
Stroma in percentage(SIP-intermediate=1)	0.55 (0.42-0.73)	**<0.001**	0.53 (0.41-0.7)	**<0.001**
Lymphocyte in percentage(LIP-high=1)	0.52 (0.39-0.68)	**<0.001**	0.52 (0.39-0.68)	**<0.001**
GUS				
T-stage(T1/T2=1)	1.1 (0.63-1.9)	0.76		
N-stage(N0=1)	1.0(0.68-1.7)	0.56		
Stage(stage1=1)	1.2 (0.89-1.6)	0.23		
Stroma in percentage(SIP-intermediate=1)	0.5 (0.37-0.69)	<0.001	0.53 (0.39-0.72)	**<0.001**
Lymphocyte in percentage(LIP-high=1)	0.65 (0.46-0.92)	<0.001	0.74(0.52-1)	0.083
EMC				
T-stage(T1/T2=1)	1.1 (0.85-1.5)	0.43		
N-stage(N0=1)	1.7 (1.1-2.4)	**0.0096**	1.8 (1.1-2.8)	0.058
Stage(stage1=1)	1.7 (1.2-2.4)	**0.0038**	1.7 (1.1-2.5)	0.065
Stroma in percentage(SIP-intermediate=1)	0.53 (0.37-0.75)	**<0.001**	0.68 (0.45-1)	**0.04**
Lymphocyte in percentage(LIP-high=1)	0.55 (0.37-0.8)	**0.0021**	0.65 (0.41-1)	**0.04**
TCGA				
T-stage(T1/T2=1)	1.5 (1-2.3)	**0.043**		
N-stage(N0=1)	1.5 (1-2.3)	**0.037**	1.3 (0.88-2)	0.17
Stage(stage1=1)	1.6 (0.95-2.7)	0.08	0.99 (0.64-1.6)	0.98
Stroma in percentage(SIP-intermediate=1)	0.34 (0.22-0.52)	**<0.001**	0.3 (0.19-0.46)	**<0.001**
Lymphocyte in percentage(LIP-high=1)	0.4 (0.26-0.6)	**<0.001**	0.33 (0.21-0.51)	**<0.001**
